# An Intelligent System for Monitoring Skin Diseases

**DOI:** 10.3390/s18082552

**Published:** 2018-08-04

**Authors:** Dawid Połap, Alicja Winnicka, Kalina Serwata, Karolina Kęsik, Marcin Woźniak

**Affiliations:** Institute of Mathematics, Silesian University of Technology, Kaszubska 23, 44-100 Gliwice, Poland; Alicja.Lidia.Winnicka@gmail.com (A.W.); Kalarcika@gmail.com (K.S.); Karola.Ksk@gmail.com (K.K.); Marcin.Wozniak@polsl.pl (M.W.)

**Keywords:** ambient intelligence, artificial neural network, skin diseases detection, pattern recognition, hybrid solutions, sensors networks

## Abstract

The practical increase of interest in intelligent technologies has caused a rapid development of all activities in terms of sensors and automatic mechanisms for smart operations. The implementations concentrate on technologies which avoid unnecessary actions on user side while examining health conditions. One of important aspects is the constant inspection of the skin health due to possible diseases such as melanomas that can develop under excessive influence of the sunlight. Smart homes can be equipped with a variety of motion sensors and cameras which can be used to detect and identify possible disease development. In this work, we present a smart home system which is using in-built sensors and proposed artificial intelligence methods to diagnose the skin health condition of the residents of the house. The proposed solution has been tested and discussed due to potential use in practice.

## 1. Introduction

Smart homes have become the key elements in the real estate market. In-Built electronics and home management systems are becoming more and more popular due to numerous advantages. They not only improve the comfort of human life, but also contribute to nature for example by saving energy. A classic example is light control in smart home environment. Another advantage, which is widely appreciated, is increased safety and protection through motion sensors or cameras attached to the alarming system which quickly prevents burglary. These are just a few important elements characterizing the trend of creation of intelligent homes. One of advantages which have important influence on humans is protection of the health of the household members. As protection, we mean counteracting to the development of disease, controlling and motivating while adhering to diets and sports. Such actions enable rapid detection of diseases and care for the correct and healthy body functioning.

Leaving the house results in exposure to the sunlight. This type of action might seem harmless, as long as we are not exposed during the strongest sun rays on hot days. In the last few years the risk of cancer has increased [1,2] and one of the most serious skin diseases is malignant tumor, i.e., melanoma. It can occur as pigmented nevus on our skin. Any skin change can mean a mutation of genes and the occurrence of melanoma, which is a type of malignant tumor. It is estimated that melanoma accounts for up to 10% of all human skin cancers, and on average, more than 1,300,000 cases are diagnosed yearly [3,4]. Each year, this number is growing rapidly—on average, about 5% compared to the previous year. Therefore a rapid detection of the disease at the initial stage increases patient’s survival prognosis. The later detection, the more difficult is the treatment. Moreover, the likelihood of metastasis is larger, and thus the survival rate is lower.

To increase the awareness of the household members about the existing disease, their state of health as well as visible signs on/in the body should be controlled. It can save their health by fast detection and treatment. A good solution that will not cause major inconveniences is to design an intelligent solution that can analyze this type of signs during other activities. Smart homes have in-built sensors that can improve control and alert quickly about the need for a visit to a specialist doctor. The awareness of people about the risk of cancer increases, which is evident from the example of education in schools, or information on the Internet, television and radio. Researchers from around the world are trying to create solutions that could be used in practice and do not cost a lot of computing power and money.

The latest research in the field of Internet of Things that can be classified as a help for health protection show a significant number of scientists focused on the analysis of people’s behavior and emotion in order to determine their health status. This type of analysis uses the information from various types of sensors, such as cameras or smart watches. One of the key examples of this type is presented in [5]. The authors focused on the use of image processing methods to help sick and elderly people. Obtained results are presented for selected artificial intelligence techniques such as decision trees, fuzzy logic or support vector machine, and as the greatest advantages are given an accuracy at 80% and low costs. Similar approaches are shown in [6,7], where the influence of emotions on these systems is measured. Sensors that record video, sounds or movements are very often used in monitoring. The retrieved data may indicate some deviations from the norm in the behavior of the household members. Information can be processed in terms of behavior analysis [8,9,10]. There are also very interesting research on sensing technologies for robotics which can assist elderly people in daily routine [11]. The use of machine learning methods requires a certain database on which the classifier will be trained. The extraction and segregation of data in smart homes were analyzed in [12]. For the purposes of verification of the proposed method, the authors collected data during 30 days, after which classifier’s accuracy was obtained at 85%. A useful tool is a smart phone that can integrate various sensors, measurement data and display them in an intelligible form for each person. The only problem is the configuration of the installed application in relation to location of the sensors or different construction of houses. An interesting solution is the automatic generation of code for the application on the android platform, which results in great flexibility of operation [13]. Another issue beside detection is the anticipation of the appearance of premature signs of diseases such as Alzhaimer [14] or the simplest application, such as assessing the state of current health and nutrition [15]. All sensors and the entire operating system that manages them or processes the obtained data requires some computing power and database to store the information. The solution is a special server or the use of cloud computing [16,17].

### Related Works

Detection of skin diseases from the processing of images is an important topic both for sensors and sensing methods but also for Computational Intelligence and image processing. There are also research on medical symptoms of skin diseases and devoted skin markers for chemical analysis. In [18] was presented how plasmacytoid dendritic cells, inflammatory dendritic epidermal cells, and Langerhans cells influence anti-viral skin defense mechanism. In [19] was discussed the diagnostic gold standard for skin diseases detection based on the analysis of auto-antibodies and mucous membranes by the use of immunofluorescence microscopy and commercialized tools. The authors presented how the antigens influence skin characterization for serological diagnosis. In [20] was discussed a chemical method to detect atopic dermatitis, psoriasis and contact dermatitis characterized by transcriptomic profiling. Potential disease was detected by the use of protein expression levels.

In [4] was presented classification of skin lesions using Deep Convolutional Neural Network. Proposed model was trained from images, using only pixels and disease labels on the input. The research presents classification in two cases: keratinocyte carcinomas vs. seborrheic keratoses and malignant melanomas vs. nevi. In [21] was proposed a technique for melanoma skin cancer detection. Authors proposed a system which combines deep learning with proposed skin lesions ensemble method. In [22] was presented optoacoustic dermoscopy model based on a function of excitation energy and penetration depth measures for skin analysis by the use of ultra imaging. It was shown how to analyze absorption spectra at multiple wavelengths for visualization of morphological and functional skin features. An approach to detect skin lesion and melanoma based on deep learning model was proposed in [23]. The authors have discussed segmentation and feature extraction in relation to the center of melanoma by Lesion Indexing Network which uses Fully Convolutional Residual Network as a main detection method. In [24] we can find an up to date review over various machine learning methods applied to skin diagnosis and diseases detection. While in [25] we can find a wide comparison of mobile apps for skin monitoring and melanoma detection. The authors have compared several apps, discussed their potential in efficient image processing form smart phone camera and sensing technologies and also presented legal aspects of ethical, quality and transparent development of apps for medical use.

Beside to the research on imge analysis methods a constant development in sensing technology is visible. In [26] an intelligent sensing system for monitoring human skin and pathogen microbiota was presented. The system was designed as a composition of nanowire and thin film metal oxide technologies. The device uses GC–MS (Gas Chromatography–Mass Spectrometry) and SPME (Solid Phase Micro Extraction) as methods of data sampling and analysis. There are also various tests of efficiency and operability measured for cameras used in skin sensing technologies. In [27] was presented a review of hyperspectral imaging one.

In this article we propose a smart sensing for detection of skin diseases resulting from changes in nevi over body area. In our system the image from a smart home camera is analyzed by the proposed methodology. The novelty of our proposal is in efficient detection and estimation of the analyzed nevus. The method first searches over the image for any potential dangerous skin changes using key point search algorithm. After the image is clustered according to their locations. Selected clusters are forwarded to the proposed neural network architecture which analyzes them for changes in skin features. Therefore by the use of the proposed novel solution the household members have a constant monitoring of their health. The solution is very easy to use, we just need to stand in front of the camera, which in our system was placed over the mirrors to enable dynamic health control within daily routine. The image is automatically forwarded to analysis and the results are presented to the household member on the connected devices he/she choses. The solution is very efficient and the tests have shown accuracy and precision rates for our idea of 82.4% and 80% respectively.

## 2. Intelligent System for Monitoring Skin Diseases

Let us assume that a smart home has its own monitoring system. Proposed type of operation is using cameras with motion sensors which collect images of the people for analysis of the skin. When these cameras are used, some specific requirements should be stated. Especially, we need very good recording quality and high resolution. Unfortunately, these parameters generate higher costs. In addition, the processing of video files will be very burdensome for the server, which will have 24 frames (identified with images) per each second acquired from one camera and in the home, there will be many of them.

Of course, this solution is possible with the assumption of a good server that can handle such amounts of the data. As an alternative solution, we suggest combining a camera with a motion sensor. In the case of a motion in a given area, photos will be taken within a few seconds (selection of this parameter should depend on the available computing power). Placing this sensor is basically simple, for example, in the vicinity of the mirrors, because we stop for a moment to look while dressing or in the morning to improve something in the hair, etc. We take into account the argument that often mirrors are placed only in the bathroom. Therefore we suggest creating a picture-taking control that can be stopped using a remote control. This solution seems to be simple and intuitive even for elderly or disabled people.

**Figure 1:**
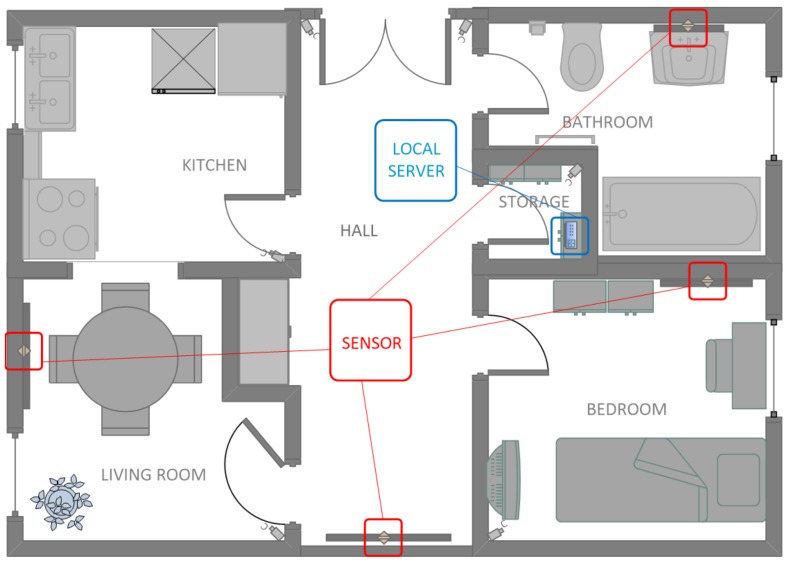
An example of a smart home visualization with the placement of sensors over mirrors in the house (the camera is activated by the motion sensor) and the location of a local data server.

A person may want to check the condition of their skin in other places (not visible to the camera), so additionally the sensor should be small and possible to be mounted on the wall or stand on the cabinet. The choice of the place depends only on the size of the equipment—the smaller the equipment, the more convenient it will be to use. In Figure 1, there is an example of a smart home model in which monitoring cameras are located and controlled by composed motion sensors. Locations have been chosen in a way that they do not interfere with any activities of the household members. Moreover, a local server placed in a small room (here, called storage) is responsible for downloading data via local wireless network from the cameras to a database stored on that server. A small disadvantage is that motion sensors which are associated with the cameras may have a short range, i.e., it would not be possible to apply this solution in places such as a living room or hall, where a house traffic could be recorded over a distance.

**Figure 2:**
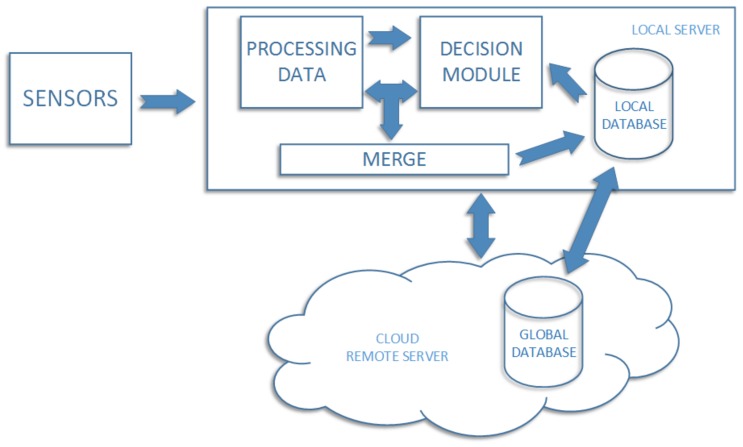
Diagram of information flow in the home management system.

In Figure 2, we present a general information flow diagram which shows how obtained information is processed in our system. At first, sensor data is downloaded and saved on the local server. Each photo is processed by the skin analysis module, after which it is analyzed by the classifier. Each image with the decision obtained from the classifier is retained in the local database. In case when the classifier result means high probability of the skin disease, the information is sent to the user immediately. There is a risk of hardware failure, which may result in data loss. To prevent such situation, the local database is synchronized with the external one, located in the cloud (called global). An additional advantage of this solution is the situation when the user installs the system at the home, but the local classifier cannot classify images because it was not trained before. In such situation cloud system starts to work as first one, since it is previously trained, standard structure of the classifier saved on the global database. In practice, after installing the system in home, the local classifier should be updated with the latest information retrained from the cloud. On the part of the contractor, there should be a classifier in the cloud, which after a certain period of time (sometimes even weeks) will be overhauled using data received from various clients. This will allow automatically updating the global classification data for each client separately using one resource.

The use of a double database means that the proposed system is resistant to failures. Of course, the loss of the global database will be much more expensive, but there is a fast possibility of recovering the data by downloading them separately from all the clients and after that combining it again into one global data. Additionally, the classifier training is in the public cloud, so this process does not burden client’s local server. Another advantage is an easy modification of this installation by adding or removing additional sensors. In a case of a large data inflow, processing support will depend on the time of saving on the server’s disk.

## 3. Skin Analysis Module

Skin diseases can manifest themselves differently, especially when the new distinctive signs are visible. These types of changes may appear slowly, which often avoids quick observation. It is particularly important to analyze any new nevus on the skin, because this can start to change, which is one of the first symptoms of skin cancer. Changes are visible and often classified as follows

- *A* (asymmetry)—any two halves of the nevus are asymmetrical;
- *B* (border)—the boundary between the healthy tissue and the nevus is fuzzy, tilted or invisible;
- *C* (color)—the nevus has different shades of color, and the border between them is irregular;
- *D* (diameter)—the diameter is greater than 6 mm;
- *E* (evolving)—there is a sudden enlargement or bulge.

However any suspicious change in nevus shape, color or structure requires particular attention.

### 3.1. Image Processing

For a given body image, it is important to locate the changes in the skin, detect all the hallmarks and classify them as a healthy skin or suspicious disease. Let us assume that the obtained image will contain large part of body. It is necessary to extract the skin and then to analyze all suspicious areas. Having an image, we are looking for areas where we can expect some important elements.

**Figure 3:**
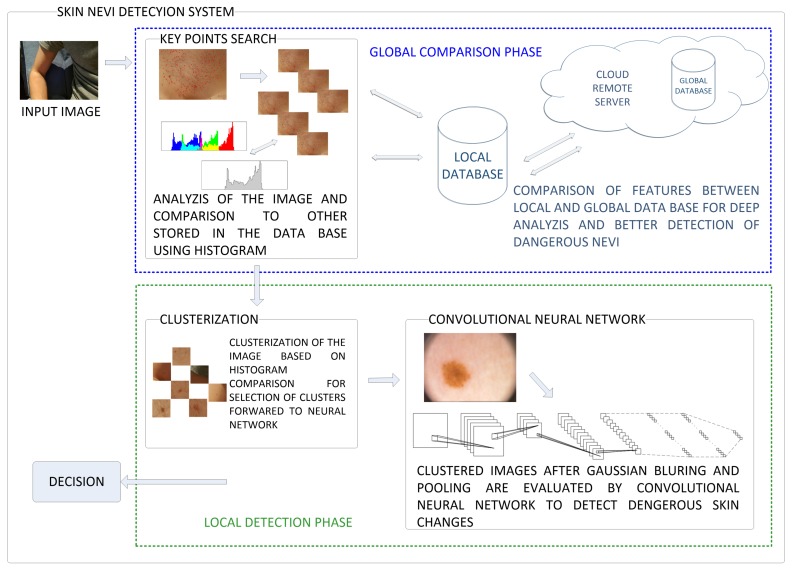
The captured image goes through the following stages of processing in the proposed system: global and local features extraction and comparison. In the first one an image is clustered into smaller parts containing suspicious symptoms over the skin. These clusters are compared by the use of histograms and selected ones are forwarded to detection phase in which Convolutional Neural Network performs evaluation of these symptoms.

**Figure 4:**
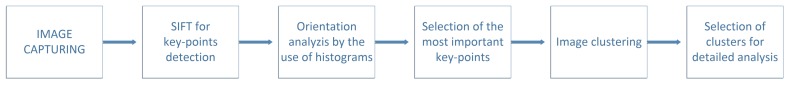
The processing of input image goes through the following stages first to locate the key-points over the image and after to select among them the most important ones which will be clustered and forwarded to the detection stage in the form of a blurred nevi image.

The obtained image *I* is colorful and two-dimensional. We should detect human skin for further analysis. The proposed approach consists of processing the image and detecting features, and then classify them. The scheme of the proposed skin nevi analysis module is presented in Figure 3. For a given image *I*, we search for key points (representing the features of the image) using the classic detection algorithm, and then each of these points will be analyzed in terms of the quality of the detection (containing a nevus or birthmark) and used to create a small image called cluster. This cluster will contain an area with a given feature for later classification by the use of Convolutional Neural Network. The process is shown in Figure 4.

### 3.2. Key Points Search

A mentioned algorithm of key points detection is called Scale Invariant Feature Transform (in short *SIFT*) [28,29]. The algorithm works on the rule of four steps, where the first one consists of detecting local extremes on different scales. Key-points are searched for depending on certain local features of the area and should be independent from scale or orientation. In addition, each point should correspond to the local extreme in the image after applying the Gauss filter (on different scales). Let us assume that the Gauss filter will be described as a continuous function in the following way

(1) $$G\left(x,y,\sigma \right)=\frac{1}{2\pi \sigma^{2}}\exp\left(-\frac{x^{2}+y^{2}}{2\sigma^{2}}\right),$$

where σ is a parameter understood as a variance. The operation of applying this filter to the given image can be described as convolution in the following way

(2) L(x,y,σ)=G(x,y,σ)·I(x,y).

Next, Difference-of-Gaussian (*DOG*) must be created for different scale *k* as

(3) D(x,y,σ)=G(x,y,kσ)−G(x,y,σ).

Detection of local extremes works on the basis of the comparison of a given point with its neighbors in the square 3×3, where the analyzed point is in the middle. It is possible to increase the size of the grid, but the used photographic equipment should take pictures of very high quality because attempts to use larger dimension resulted in the selection of non-discriminating elements. A given point can be considered as an important if its value is maximal or minimal in relation to the neighbors. In the next step, points are localized by interpolating previously found extremes using Taylor series expansion as

(4) $$D\left(\bar{x}\right)=D+\frac{\partial D^{T}}{\partial \bar{x}}\bar{x}+0.5{\bar{x}}^{T}\frac{\partial^{2}D}{\partial {\bar{x}}^{2}}\bar{x},\;\mathrm{where}\;\bar{x}={\left(x,y,\sigma \right)}^{T}.$$

In addition, unstable points are rejected due to the low contrast value which is verified by the following function

(5) $$D\left(\overset{˘}{x}\right)=D+0.5\frac{\partial D^{T}}{\partial x}\overset{˘}{x},\;\mathrm{where}\;\overset{˘}{x}=-\frac{\partial^{2}D^{-1}}{\partial {\bar{x}}^{2}}\frac{\partial D}{\partial \bar{x}}.$$

After, points on the edges are removed if the following condition is fulfilled

(6) $$\det H<0\;\mathrm{or}\;\frac{\mathrm{tr}(H)}{\det(H)}<\frac{{\left(r+1\right)}^{2}}{r},$$

where *r* is a given parameter of a neighborhood and tr(·) is a trace of a given Hessian matrix defined as

(7) $$\left[\begin{array}{cc} \partial_{xx}^{2}D & \partial_{xy}^{2}D\\ \partial_{yx}^{2}D & \partial_{yy}^{2}D \end{array}\right].$$

The third step is the orientation of all remaining points from Gaussian images. It is done by using gradient module m(x,y) and orientation ϕ(x,y) defined as

(8) $$m\left(x,y\right)=\sqrt{{\left(L\left(x+1,y\right)-L\left(x-1,y\right)\right)}^{2}+{\left(L\left(x,y+1\right)-L\left(x,y-1\right)\right)}^{2}},$$

(9) $$\phi \left(x,y\right)=\arctan\left(\frac{L(x,y+1)-L(x,y-1)}{L(x+1,y)-L(x-1,y)}\right).$$

**Figure 5:**
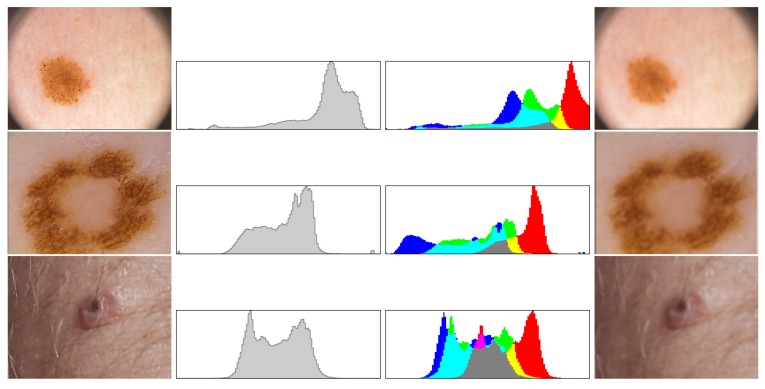
Each cluster is analyzed by the use of histograms (in gray and full color), which are compared to other images stored in the data base system before blurring operation. In the rows 1–3 we see examples of suspicious nevi, both histograms and blurred cluster that will be forwarded to evaluation phase.

**Figure 6:**
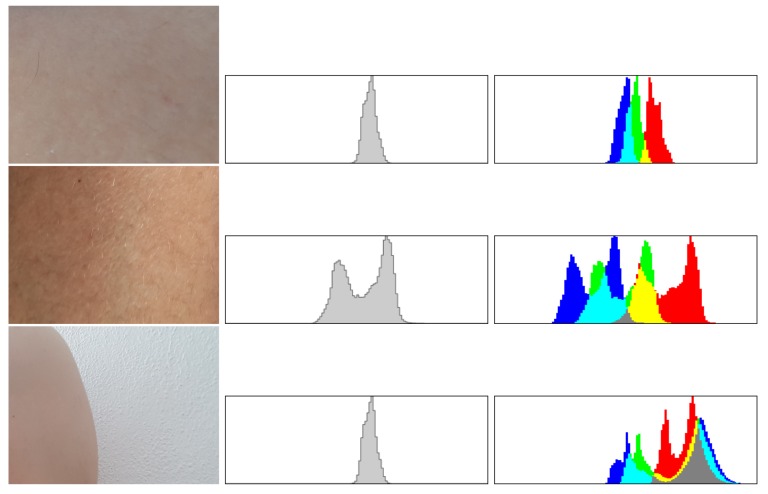
If the analysis of histograms shows that the cluster presents normal skin the images is not forwarded to evaluation phase. In the rows 1–2 we see examples of regular healthy skin and in the last one a fragment of a skin over a wall. The images show a cluster and both histograms, since there are no suspicious nevi therefore all without blurring necessary for evaluation phase.

**Figure 7:**
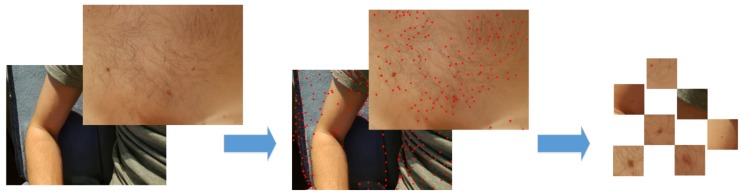
The process of creating clustered image files with nevi for classification. The original image is processed by the use of SIFT method which shows potential locations of key-points. After clusters are composed, and each of them is evaluated and cropped to show the suspicious nevus in the best possible way to make the evaluation the next phase more efficient.

Above formulas allow creating a histogram of orientation values weighted by calculated module and function G(|(x+i,y+j)−(x,y)|,σ), where (i,j)∈{−1,0,1}. Further, the greatest value in histogram is selected, and all values above 80% of selected maximum are treated as a feature orientation of this point. This action caused that key points are independent from rotation. The last step generates descriptors for this points. A certain neighborhood of the point is taken. Then all elements of neighborhood are divided into smaller, even areas. In each area, resultant gradient modules are calculated, which allows to define a descriptor. Sample images of healthy skin and nevi with their histograms in gray and color scale are presented in Figure 5 and Figure 6, while the procedure to select clusters is presented in Figure 7.

### 3.3. Selection of Found Key Points

All the points found by using SIFT will be located on the entire image, so they will adhere to various skin features. Imagine the simplest and the most likely situation when such a picture is taken in front of a mirror. The householder will not occupy the entire picture, but only a part of it. The rest of the area will concern the elements of home furnishings. The points that indicate this objects should be removed and not taken into account.

The easiest way is to take several photos earlier (with different lighting), find key points and save their coordinates with colors. In the case when the same point appears on the new image, it will be automatically deleted. We also remove these which colors do not fit the palette colors in the data base since we assume these may reflect new clothing or jewelery. Additionally border lines and other long, thin shapes are removed. Moreover, there is a chance that a given point will be characterized by a certain part of the body such as hair, eyes or lips. The exact elimination of these parts is not so desirable, because at a later stage, the classifier will decide on the validity of the element. However, in order to improve the operation of the entire system, when the number of images is smaller, the computational power needed to process them is also lower.

### 3.4. Creation of Suspicious Clusters

After the previous selection of points, each of them can be presented in the form of a cluster. The cluster will be called a small, separate area from the original image containing the given point. An intuitive example may be the point of the nevus, where the cluster is an image which takes a full picture of the nevus. In practice, the idea is to create a large area with a given point, which is processed to extract the localized object and trim to its size. Assume that the key point is at position (x,y). A new bitmap of size 15%w×15%h is created (where *w*, *h* correspond respectively to the width and the height of the original image). In the center is located the key-point. Then, we change all colors to gray-scale by averaging the value of each component of a given pixel (i,j) as

(10) $$C\left(i,j\right)=\frac{R(i,j)+G(i,j)+B(i,j)}{3},\;\mathrm{where}\;C\left(i,j\right)=\left\{R\left(i,j\right),G\left(i,j\right),B\left(i,j\right)\right\}.$$

After, we modify the images by applying gamma correction and increasing the contrast to eliminate unnecessary, small elements in the image. Modifications for a given point can be defined as

(11) $$C\left(i,j\right)=255\cdot {\left(\frac{C(i,j)}{255}\right)}^{\gamma },$$

where γ is a coefficient of correction (γ≠0). The second formula is described by

(12) $$C\left(i,j\right)=\left(\frac{259(\omega +255)}{255(259-\omega )}\left(C\left(i,j\right)-128\right)+128\right)\%255,$$

where ω is a parameter called level of contrast. After applying the above formulas to each pixel, the image obtained is much simplified. Taking the key point, we count all its neighbors as having the same color in a recursive manner. If the number of counted pixels is greater than 30%w·h, the key point is removed. Otherwise, the image is cropped relatively to the pixels that have been counted. It is easy to see if the resulting cluster is useless due to the filtering, so each of these pixels is replaced according to the original image. Additionally, in order to unify the dimensions of clusters, all images are minimized/extended to the same dimension given by the user. This process is presented in Figure 7.

#### Convolutional Neural Network

Convolutional neural networks are structures inspired by information transfer between neurons, which are basic units in the brain. Their operation is similar to classic artificial networks, so the structure itself in general description is the same. It is composed of several types of layers, each of them contains neurons. Neurons in one layer are connected with other units in the previous and next layer (if they are composed in the structure), and each connection is burdened with a weight. In the initial operation of the network, weights are selected in random way and modified during the training process.

**Figure 8:**
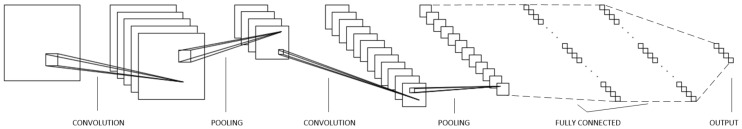
Sample scheme of the Convolutional Neural Network composed to process clustered images of suspicious skin nevi.

The main difference between the classic network and this one is the received information, which forces a certain layer structure (see Figure 8). In the classical approach, the inputs are vectors composed of information saved as numerical values. Here, images are input data. The whole idea is inspired by cells in the primary cortex [30,31]. The first layer is called convolutional, which is responsible for extracting features from the image using an image convolution operation. To describe it, let us define a filter $\omega_{ab}$ which is a matrix of size 3×3 and ab is the position of the center point in the matrix (the position is related to original image). Using this position, this filter can be moved over the image by step *S*. This layer uses $\omega_{ab}$ to modify the image in a way to isolate or highlight certain important features by adding certain pixels to local neighbors multiplied by a value called the kernel and described as mentioned before $\omega_{ab}$. In short description, in this layer, the image is only modified relatively to the given filter. Gaussian blur is one of the filters that distorts the image and is described as

(13) $$\omega_{ab}=\frac{1}{16}\left[\begin{array}{ccc} \left(a-1,b-1\right) & \left(a-1,b\right) & \left(a-1,b+1\right)\\ \left(a,b-1\right) & \left(a,b\right) & \left(a,b+1\right)\\ \left(a+1,b-1\right) & \left(a+1,b\right) & \left(a+1,b+1\right) \end{array}\right]=\frac{1}{16}\left[\begin{array}{ccc} 1 & 2 & 1\\ 2 & 4 & 2\\ 1 & 2 & 1 \end{array}\right].$$

Another important filter is emboss which modifies a given pixel by replacing it with a back light or shade depending on the original one and the kernel is defined as

(14) $$\omega_{ab}=\left[\begin{array}{ccc} \left(a-1,b-1\right) & \left(a-1,b\right) & \left(a-1,b+1\right)\\ \left(a,b-1\right) & \left(a,b\right) & \left(a,b+1\right)\\ \left(a+1,b-1\right) & \left(a+1,b\right) & \left(a+1,b+1\right) \end{array}\right]=\left[\begin{array}{ccc} -2 & -1 & 0\\ -1 & 1 & 1\\ 0 & 1 & 2 \end{array}\right].$$

The second type of layer is called pooling one. It’s main task is to reduce the incoming image by selecting certain pixels. The idea is similar to the operation of convolutional layer, with the difference that on each matrix 3×3 from input image, one pixel is created to replace the previous 9. Let us denote the matrix of pixels as $P=[p_{ij}]$ where i,j∈{1,2,3}, then the returning pixel can be selected as the maximum element in matrix or in terms of a certain parameter like one of the RGB value or the average color as

(15) $$\mu_{1}\max_{i,j\in \{1,2,3\}}\left(R(p_{ij})\right)+\mu_{2}\max_{i,j\in \{1,2,3\}}\left(G(p_{ij})\right)+\mu_{3}\max_{i,j\in \{1,2,3\}}\left(B(p_{ij})\right),$$

where $\mu_{1}$, $\mu_{2}$ and $\mu_{3}$ are scales that attenuate the importance of a given color only if $\sum_{i=1}^{3}\mu_{i}=1$.

For the 3×3 dimension matrix, the output matrix from this layer will be three times smaller. The last type of layers is called fully-connected and their operation is almost identical to the classical neural network. The construction of this layer can be separated into smaller elements resembling the idea of hidden and output layers in classic understanding. At the input to this layer, there is a small image. Each pixel must be described as a numerical value and as a result of such representation, the vector with numerical values is formulated. Next, a column of neurons is created where the number of units is identified with the number of pixels form the input image. Operation of the neuron can be described as a following function

(16) $$y_{k}=\phi \left(\sum_{j}^{n}w_{jk}x_{j}\right),$$

where $y_{k}$ is the output from neuron, $x_{j}$ is the output from neuron in the previously layer, $w_{jk}$ is the weight on the connection between *j*-th and *k*-th neurons and ϕ(·) is the activation function defined as

(17) ϕ(x)=tanh(x),

which normalizes the value of the sum to the range 〈−1,1〉.

Fully-connected layer can be composed of many smaller layers with neurons, where in the last one are output neurons (their number is defined depending on desired classification results).

Of course, such structure is useless before training, and one of the most popular algorithms for this purpose is back-propagation algorithm (which modifies the weights to minimize the error of the whole structure) [30,32]. To describe the actions, some markings should be defined like error function f(·) and error at the end of the network as $\frac{\partial f}{\partial y_{ij}^{l}}$. In addition, we specify the output from a particular neuron at position (i,j) in *l*-th layer as $y_{k}=\frac{\partial f}{\partial y_{ij}^{l}}$. The training algorithm is using chain rule defined as follows

(18) $$\frac{\partial f}{\partial \omega_{ab}}=\sum_{i=0}^{N-m}\sum_{j=0}^{N-m}\frac{\partial f}{\partial x_{ij}^{l}}\frac{\partial x_{ij}^{l}}{\partial \omega_{ab}}=\sum_{i=0}^{N-m}\sum_{j=0}^{N-m}\frac{\partial f}{\partial x_{ij}^{l}}y_{\left(i+1)(j+b\right)}^{l-1}.$$

Equation (18) allows to define error formula for the current layer in the following manner

(19) $$\frac{\partial f}{\partial x_{ij}^{l}}=\frac{\partial f}{\partial y_{ij}^{l}}\frac{\partial y_{ij}^{l}}{\partial x_{ij}^{l}}=\frac{\partial f}{\partial y_{ij}^{l}}\frac{\partial \left(\phi (x_{ij}^{l})\right)}{\partial x_{ij}^{l}}=\frac{\partial f}{\partial y_{ij}^{l}}\phi^{\prime }\left(x_{ij}^{l}\right).$$

Based on Equations (18) and (19), it can be seen that the algorithm uses gradient technique to minimize error value, so it can be also done for calculating error in convolutional layer (this rule cannot be applied to pooling layer, which does not take part in training process) as

(20) $$\frac{\partial f}{\partial y_{ij}^{l-1}}=\sum_{a=0}^{m-1}\sum_{b=0}^{m-1}\frac{\partial f}{\partial x_{\left(i-a)(j-b\right)}^{l}}\frac{\partial x_{\left(i-a)(j-b\right)}^{l}}{\partial y_{ij}^{l-1}}=\sum_{a=0}^{m-1}\sum_{b=0}^{m-1}\frac{\partial f}{\partial x_{\left(i-a)(j-b\right)}^{l}}\omega_{ab},$$

what gives the formula for error on previous layer determined as

(21) $$\frac{\partial x_{\left(i-a)(j-b\right)}^{l}}{\partial y_{ij}^{l-1}}=\omega_{ab}.$$

## 4. Experimental Section

**Figure 9:**
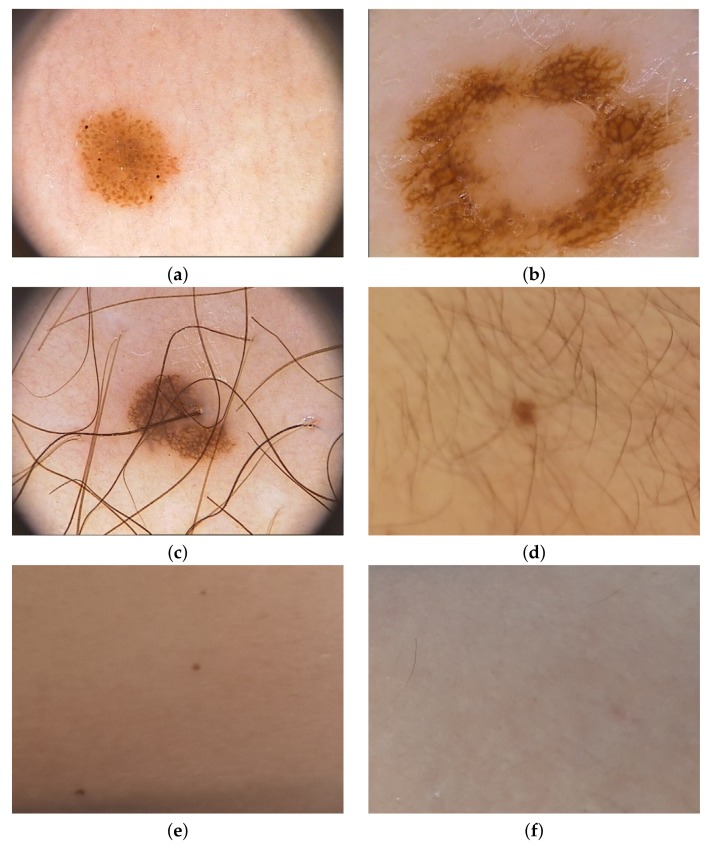
Selected samples used in training process—(**a**–**c**) come from PH2 Dataset, and (**d**–**f**) were obtained from the method described in this paper.

To check the operation of the proposed solution, we first tested the operation of the proposed method for creating images from photos taken in different places in six houses, where we mounted sample sensors of our system to collect new images from the household members. After we have added an open database PH2 Dataset [33] to extend our collection. PH2 Dataset contains images from the Dermatology Service of Hospital Pedro Hispano (Matosinhos, Portugal) presenting nevi in 8-bit RGB color images with a resolution of 768 × 560 pixels. PH2 Dataset contains 200 dermoscopic images of melanocytic lesions, where 80 are common nevi, 80 are atypical nevi, and 40 are melanomas. Our new images are in resolution 800 × 600, where we have collected 73 images of nevi. Figure 9 presents samples from both data sets.

The main purpose of classification is to make decision about the input data and assign to this the value from decision set $\left\{\alpha_{1},\cdots ,\alpha_{n}\right\}$. It means that $\alpha_{i}$ is decision class for *i* from 1 to *n*. For every element *x* in the input database, we verify whether the decision was right. That is, if $\alpha \left(x\right)=\alpha_{i}$. The test result is *negative* only if $\alpha \left(x\right)\neq \alpha_{i}$. Otherwise the test result is *positive*. We assume that the condition $\alpha \left(x\right)\neq \alpha_{i}$ is the null hypothesis and the opposite to this, $\alpha \left(x\right)=\alpha_{i}$ we call an alternative hypothesis. Let *f* be the classifier and take one of decision classes $\alpha_{i}$. Then test results can be described by 4 parameters

- TP—True Positive, when $\alpha \left(x\right)=\alpha_{i}$ and $f\left(x\right)=\alpha_{i}$,
- TN—True Negative, when $\alpha \left(x\right)\neq \alpha_{i}$ and $f\left(x\right)\neq \alpha_{i}$,
- FP—False Positive, when $\alpha \left(x\right)\neq \alpha_{i}$ and $f\left(x\right)=\alpha_{i}$, what means that the type I error was made—the null hypothesis was rejected even though it was correct,
- FN—False Negative, when $\alpha \left(x\right)=\alpha_{i}$ and $f\left(x\right)\neq \alpha_{i}$, what means that the type II error was made—the null hypothetis was not rejected even though it was not correct.

For our model we have two decisions: the image presents the nevus which does not cause dangerous effects to health and opposite which means the nevus is dangerous and might have consequences to health. Therefore our hypothesis $h_{0}$ is to reject that the nevus is dangerous.

**Figure 10:**
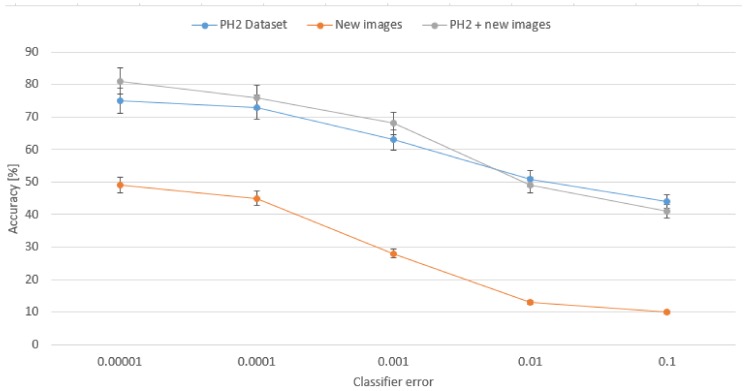
Dependency between the classifier’s error and the average accuracy presented for different assumed error in the classifier for all three configurations of the input data collection.

The scores we obtained in our research are shown in Table 1. We can see that the best results are achieved for merged datasets, where precision is 80% and accuracy 82.4%. These results are almost twice better in comparison to single set of new images. We can also see that for single PH2 dataset accuracy was improved of about 3% while precision decreased of 10%. This is a result of extended set of input images, since for a single origin the classifier was trained on a specific nevi detection so precision was higher while for new images new possibilities came so the precision is lowered. On the other hand, what is more important, the accuracy rate is the highest for merged sets. Figure 10 presents how the accuracy rate changes for different assumed error from our classifier for all three configurations of the input data collection. Analysis of this chart confirms that extended data collection improves the classifier for more efficient detection of dangerous nevi on the skin.

**Figure 11:**
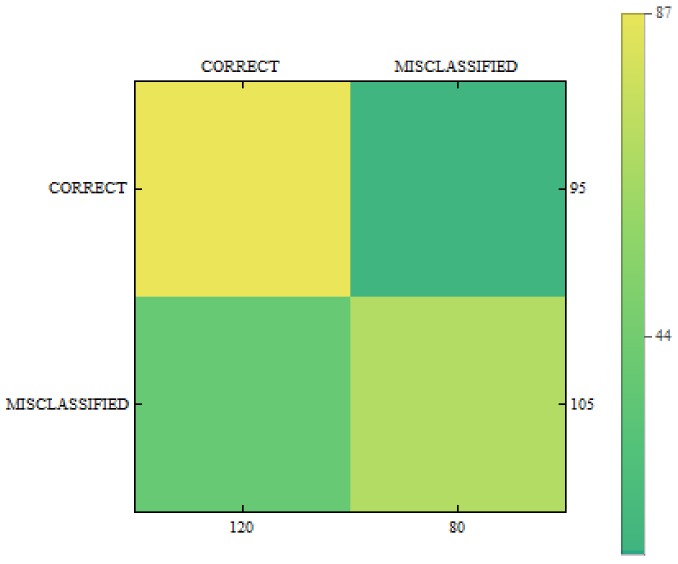
Confusion matrix for measurements obtained by the classifier using PH2 Dataset only.

**Figure 12:**
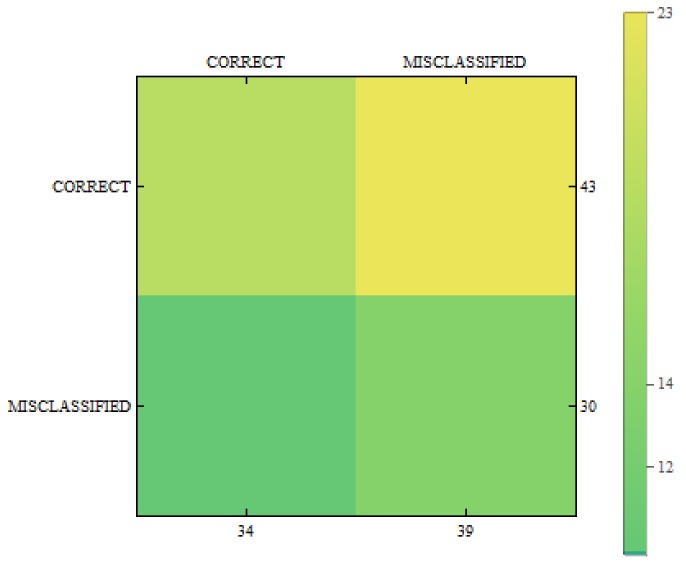
Confusion matrix for measurements obtained by the classifier using created images from the proposed method only.

**Figure 13:**
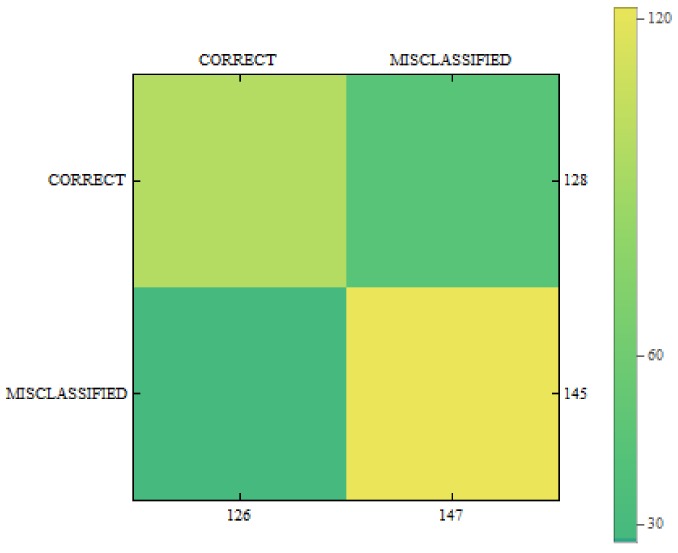
Confusion matrix for measurements obtained by the classifier using PH2 Dataset and created images from the proposed method.

In Figure 11, Figure 12 and Figure 13 we can see confusion matrices for classification results. If we compare these charts we will see how decision classes change for each of training configurations. The best recall rates are visible for merged collections from both datasets. While in case of single training sets we can see that false positive and false negative rates influence the overall decision classes of the classifier. In Figure 11 the relation between decision classes show that we had more false positives than false negatives. In Figure 12 we see that using only new images caused the highest rates of false positive, while true positives and true negatives were at similar level but still lower. In Figure 13 we see that merged collections of PH2 Dataset and our new images improved training of the proposed classifier to reach the highest rate for true positive. At the same time, true negatives reached also good level for efficient classification. In this case, both false decision classes levels were lowered to minimum so that the classifier could reach good overall accuracy of 82.4% and precision of 80%.

We have also checked whether the obtained results depend on one or more factors. For this purpose, the statistical analysis of variance (ANOVA with α=0.05) was used and the scores are presented in Table 2. Comparing F-ratio values we see that merged data sets show the highest dispersion among results. Results for single data sets are much lower, while the result for our data set is the lowest among all. These results mean that merged data sets give dispersion among the results, what is obvious since in our data set we have only a few people while in PH2 there are examples from various people. These are confirmed for F-ratios for each of the sets alone. Since in our data there are the highest similarities among samples the dispersion is the lowest. This show that introduction of new images extends dependencies on various factors, which is true since in our data set we have various nevi which show mostly healthy skin. On the other hand without our new dataset the proposed classifier was working worse which was visible in lower accuracy presented in confusion matrices in Figure 11, Figure 12 and Figure 13. Therefore we assume that merging of these two collections extends dependencies between decisions and input images but improves the overall efficiency of the proposed classifier.

### Conclusions

This research has shown that the proposed classification method works well. We were able to train our system to reach overall accuracy of 82.4% and precision of 80.4%, which is very good result. Comparison to the results from other similar solutions is presented in Table 3. Analyzing the table we see that our method is not the best, since, for example, the Circular center + Support Vector Machine [34] achieved 94% accuracy but on the other hand, our solution has beaten other methods based on Support Vector Machine alone by reaching higher values. This comparison shows that our method works well, but there is still some room for improvements, i.e., by the use of better quality cameras the images would have higher resolution what would definitely improve the results. On the other hand, our system is easy to use since it is able to monitor possible skin changes during daily routine at very low costs. Household members can make evaluations of nevi just by using any mirror. The procedure is simple, since mounted cameras collect images and by the use of the proposed method select body are with clustered areas of the skin. The evaluation is using proposed methodology which compares clustered skin outlook with images of nevi stored in the database.

## 5. Final Remarks

Frequent control of any symptoms which may signal potential disease is a crucial condition for efficient medicine. During our life we are often so busy with family, work, running a house, etc. that we are leaving aside medical examinations. Therefore an automated support is a solution which can help us to maintain our health. This kind of support shall be installed in a place where we feel good and where the evaluation of the symptoms can be the most efficient. Therefore to solve these issues we propose a smart home system. At home we feel safe and proposed installation of cameras over mirrors make the use of this system very easy. Proposed methodology collects information about skin changes and if any potential risk occurs informs the users.

Experimental research have shown high efficiency and gives good start for further development. It is possible to extend the system ability to examine not only nevi on the skin but other features of our bodies. Proposed architecture can be also extended to automatically contact the doctor in case of emergency. It is also possible to develop a portable version of this solution which we can take for a holiday or a business trip.

## Figures and Tables

**Table 1:** Quality measures of classifier trained only for the data from PH2 Dataset, only for obtained new images from proposed method and merged both dataset.

	PH2 Dataset	New Images	PH2 + New Images
Accuracy (Acc)	0.795	0.493	0.824
Precision	0.915	0.465	0.804
Sensitivity (Se)	0.725	0.588	0.817
Specificity (Sp)	0.7	0.41	0.829
Fall-out	0.1	0.589	0.17
False negative rate	0.275	0.411	0.182
Negative predictive rate	0.685	0.533	0.841
False discovery rate	0.084	0.534	0.195
F-measure	0.809	0.519	0.811

**Table 2:** Statistical results for the ANOVA test.

	**PH2 Dataset**				
	**DF**	**SumOfSq**	**MeanSq**	**FRatio**	**PValue**
Model	2	1.031	0.515	2.794	0.0635727
Error	198	36.531	0.184		
Total	200	37.562			
	**New images**				
	**DF**	**SumOfSq**	**MeanSq**	**FRatio**	**PValue**
Model	2	2.717	1.358	2.371	0.100723
Error	71	40.687	0.573		
Total	73	43.405			
	**PH2 + new images**				
	**DF**	**SumOfSq**	**MeanSq**	**FRatio**	**PValue**
Model	2	2.295	1.147	6.187	0.0023566
Error	271	50.259	0.185		
Total	273	52.554			

**Table 3:** Comparison of results to other methods.

Year	Method	No. of Images	Evaluation Metric
2017	Fully Convolutional Residual Network [35]	900 training, 300 testing	Se = 0.547, Sp = 0.931, Acc = 0.85
2016	Support Vector Machine [36]	408	Se = NA, Sp = NA, Acc = 0.743
2016	Circular center + Support Vector Machine [34]	NA	Se = 0.94, Sp = 84, Acc = 0.93
2016	Geodesic active contour [37]	NA	Se = 0.912, Sp = 0.958, Acc = 0.94
2015	High level features [38]	NA	Se = 0.835, Sp = 0.813, Acc = 0.811
2013	Thresholding+Improved Dynamic Programming Asymmetry [39]	NA	Se = 0.882, Sp = 0.931, Acc = NA
Proposed	Clustered key-points + Convolutional Neural Network	73	Se = 0.817, Sp = 0.829, Acc = 0.824
